# Automated Motion Analysis of Bony Joint Structures from Dynamic Computer Tomography Images: A Multi-Atlas Approach

**DOI:** 10.3390/diagnostics11112062

**Published:** 2021-11-07

**Authors:** Benyameen Keelson, Luca Buzzatti, Jakub Ceranka, Adrián Gutiérrez, Simone Battista, Thierry Scheerlinck, Gert Van Gompel, Johan De Mey, Erik Cattrysse, Nico Buls, Jef Vandemeulebroucke

**Affiliations:** 1 Department of Radiology, Vrije Universiteit Brussel (VUB), Universitair Ziekenhuis Brussel (UZ Brussel), 1090 Brussels, Belgium; Adrian.Gutierrez@uzbrussel.be (A.G.); Gert.VanGompel@uzbrussel.be (G.V.G.); johan.demey@uzbrussel.be (J.D.M.); Nico.Buls@uzbrussel.be (N.B.); jefvdmb@etrovub.be (J.V.); 2 Department of Electronics and Informatics (ETRO), Vrije Universiteit Brussel (VUB), 1050 Brussels, Belgium; jceranka@etrovub.be; 3 IMEC, Kapeldreef 75, B-3002 Leuven, Belgium; 4 Department of Physiotherapy, Human Physiology and Anatomy (KIMA), Vrije Universiteit Brussel (VUB), Vrije Universiteit, 1090 Brussel, Belgium; luca.buzzatti@vub.be (L.B.); Erik.Cattrysse@vub.be (E.C.); 5 Department of Neurosciences, Rehabilitation, Ophthalmology, Genetics, Maternal and Child Health, Campus of Savona, University of Genova, 17100 Savona, Italy; simone.battista@edu.unige.it; 6 Department of Orthopaedic Surgery and Traumatology, Vrije Universiteit Brussel (VUB), Universitair Ziekenhuis Brussel (UZ Brussel), 1090 Brussels, Belgium; Thierry.Scheerlinck@uzbrussel.be

**Keywords:** dynamic CT, motion analysis, musculoskeletal imaging, registration, segmentation, multi-atlas segmentation

## Abstract

Dynamic computer tomography (CT) is an emerging modality to analyze in-vivo joint kinematics at the bone level, but it requires manual bone segmentation and, in some instances, landmark identification. The objective of this study is to present an automated workflow for the assessment of three-dimensional in vivo joint kinematics from dynamic musculoskeletal CT images. The proposed method relies on a multi-atlas, multi-label segmentation and landmark propagation framework to extract bony structures and detect anatomical landmarks on the CT dataset. The segmented structures serve as regions of interest for the subsequent motion estimation across the dynamic sequence. The landmarks are propagated across the dynamic sequence for the construction of bone embedded reference frames from which kinematic parameters are estimated. We applied our workflow on dynamic CT images obtained from 15 healthy subjects on two different joints: thumb base (*n* = 5) and knee (*n* = 10). The proposed method resulted in segmentation accuracies of 0.90 ± 0.01 for the thumb dataset and 0.94 ± 0.02 for the knee as measured by the Dice score coefficient. In terms of motion estimation, mean differences in cardan angles between the automated algorithm and manual segmentation, and landmark identification performed by an expert were below 1°. Intraclass correlation (ICC) between cardan angles from the algorithm and results from expert manual landmarks ranged from 0.72 to 0.99 for all joints across all axes. The proposed automated method resulted in reproducible and reliable measurements, enabling the assessment of joint kinematics using 4DCT in clinical routine.

## 1. Introduction

Musculoskeletal (MSK) conditions are a leading cause of disability in four of the six World Health Organization regions [1] and a major contributor to years lived with disability (YLD) [2]. MSK diseases affect more than one out of every two persons in the United States age 18 and older and nearly three out of four age 65 and older [3]. For instance, patellar instability, which is a disease where the patella bone dislocates out from the patellofemoral joint, accounts for 3% of all knee injuries [4]. Patients with this condition can have debilitating pain, which can limit basic function, and develop long term arthritis overtime. Understanding the complexity of such conditions and improving the results of therapeutic interventions remains a challenge. Combining kinematic information of joints with detailed analysis of joint anatomy can provide useful insight and help therapeutic decision making. X-ray imaging techniques and their quantitative analysis are helpful to better understand and manage some MSK conditions, but the 2D nature of the images make detailed kinematic analysis challenging [5]. Dynamic computer tomography (4D-CT) enables acquisition of a series of high temporal-resolution 3D CT datasets of moving structures. Various phantom studies [6,7,8,9] demonstrated the validity and feasibility of dynamic CT for evaluating MSK diseases. Several patient studies have been conducted investigating different joint disorders of the wrist, knee, hip, shoulder and foot [10,11,12]. However, the accurate and reproducible detection of joint motion or subtle changes over time in clinical routine requires image analysis procedures such as image registration. This refers to the estimation of a spatial transformation which aligns a reference image and a corresponding target image.

Currently, few computer-aided diagnostic tools are available for dynamic MSK image data analysis, thus limiting the clinical applicability of quantitative motion analysis from these images. Reasons for this include the complexity and heterogeneity of the musculoskeletal system and the associated challenges in motion estimation of these structures. MSK structures can move with respect to each other, and motion can therefore not be assessed using a global rigid registration. Moreover, in most applications of dynamic MSK imaging, the piece-wise rigid motion of the individual bones is of primary interest for extracting kinematic parameters. The principal challenges for non-rigid registration are the magnitude and complexity of osteoarticular motion, often also including sliding structures, leading to poor accuracies or implausible deformation [13]. Block matching techniques have been proposed to improve robustness [14,15]. Several authors have proposed methods to account for sliding motion [16,17], but most rely on prior segmentations of bones of interest. Motion estimation of MSK structures is therefore commonly performed using prior manual segmentations of the bony structures, limiting registration to a region of interest and obtaining individual bone motion to facilitate estimation of kinematics [6,8]. However, manual bone segmentation is labor intensive and hinders application in clinical routine.

D’Agostino et al. [18] made use of image registration in estimating kinematics of the thumb to study the Screw-home mechanism. They investigated extreme positions (i.e., maximal Ex–Fl and maximal Ab–Ad) by means of an iterative closest-point algorithm. Their approach required manual segmentations of each bone for each position to generate 3D surface models. Such an approach can be labor intensive when analyzing dynamic sequences of multiple time frames or bone positions. Furthermore, the quantitative description of joint kinematics requires the reconstruction of the bone positions and orientation relative to a laboratory reference frame [19]. Skeletal anatomic landmarks help to provide what is known as bone-embedded reference frames. This determines the estimated motion of the joints in relation to anatomical axes defined on the bones. The manual identification of these anatomical landmarks on the CT images can also be a labor-intensive step. A few algorithms for automatic localization of skeletal landmarks have been proposed in literature [20,21,22]. Techniques based on machine learning algorithms which learn distinctive image features on annotated data have also been presented [22]. These techniques usually require a significant amount of annotated data to yield good results. In general, most of these approaches detect geometrical features that match the shape properties of these landmarks [20,23]. However, none of these approaches have been applied for the computation of kinematics from dynamic images.

In this work, we propose an automated framework for motion estimation of bony structures obtained from dynamic CT acquisitions. Changes in joint functionality are of diagnostic importance, the proposed automated workflow can help in quantitatively monitoring joint health as well as the impact of therapeutic interventions.

## 2. Materials and Methods

### 2.1. Subject Recruitment

After approval from our institution’s Medical Ethics Committee (B.U.N 143201733617) and written informed consent, 15 healthy volunteers (7 females, 8 males) were recruited to participate in this dynamic CT study. Ages of participants ranged from (22 to 36). Five subjects (3 females, 2 males) had a CT scan of the thumb, and 10 subjects (4 females, 6 males) had a CT scan of one of the knees. To be eligible for the study, participants should not have reported joint pain in the previous 6 months prior to the study.

### 2.2. CT Acquisitions

**Figure 1:**
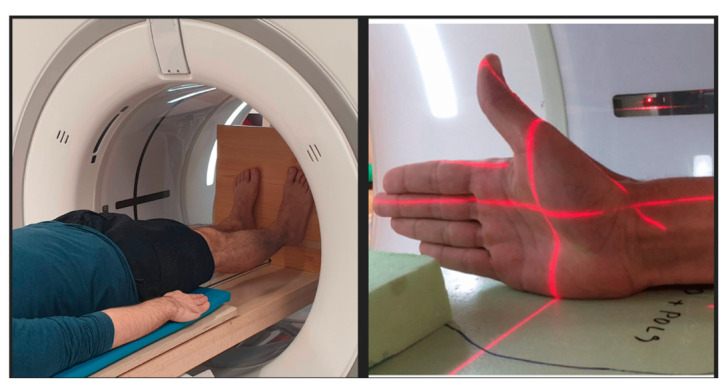
The figure shows the positioning in the gantry of the CT.

All images were acquired with a clinical 256-slice Revolution CT (GE Healthcare, Waukesha, WI, USA). The dynamic acquisition protocol consisted of low-dose images (effective dose < 0.02 mSv) obtained in cine mode. Volunteers were instructed to perform cyclic joint movements: opposition-reposition movement of the thumb (*n* = 5) and flexion-extension of the knee (*n* = 10). Static scans were also acquired of each joint without motion (Figure 1). Thumb base images were acquired with the patient sitting with a 90-degree flexed elbow, with the thumb directed upwards and the forearm in a neutral rotation. Images of the knee were acquired in full extension. The dynamic scans were acquired with a tube rotation time of 0.28 s and a total dynamic acquisition time of 6 s. This generated 15 timeframes, each composed of a 3D CT dataset. Videos of the dynamic images are available as Supplementary Data (Video S1 and S2). Details of the scan parameters are shown in Table 1. In each dynamic dataset, an image with the joint in a position similar to the static scans was selected as reference image. The selected reference image served as the input to the multi-atlas segmentation step.

### 2.3. Atlas Dataset

Atlases of the thumb base and knee were created based on the static CT scan datasets. Manual bone segmentations were performed in collaboration with an expert in bone anatomy using ITKSnap’s [24] active contour mode, followed by morphological operations and manual refinement. The patella, femur and tibia were segmented for the knee images. First, metacarpal bone and the trapezium were segmented for the thumb base. For each joint we created two separate left and right atlases. As the knee datasets were obtained with both legs in the gantry, we used an automated post-processing step for axis of symmetry detection and splitting, to separate the left from the right sides. For each dataset, a total of 9 anatomical landmarks were manually identified on the bones of interest by three expert readers. The expert readers had varying levels of expertise and training. “Reader 1” was a physiotherapist and musculoskeletal radiology research fellow with 6 years of experience, “reader 2” was an orthopedic surgeon with 30 years of experience and “reader 3” was an orthopedic surgeon specialized in hand, wrist and upper limb pathology with 4 years of experience. The mean of landmarks identified by all readers were used in the creation of the atlas anatomical landmarks for the automated algorithm.

### 2.4. Multi-Atlas Segmentation

The multi-atlas segmentation (MAS) consisted of a three-step process: (1) a pairwise registration of the image to be segmented (reference image) to the set of atlases to find optimal transformations that align each atlas to the reference image, (2) the propagation of the atlas labels onto the reference image using the corresponding transformations from step 1, and (3) a fusion step which combines all labels into a single final segmentation.

The pairwise registration step can be mathematically represented by the optimization problem below

(1) $$\hat{\mu }=\arg\underset{\mu }{\min}C\left(f\left(x\right),g_{n}\left((T_{\mu }\left(x\right)\right)\right)$$

where *f* represents the reference image to be segmented, *g_n_* is the individual atlas images and *x* is the spatial coordinate over the image. *T* is the sought spatial transformation with parameters μ which aligns the two images. The cost function *C* is composed of a similarity metric and (in the case of deformable registration) a regularization penalty. 

We implemented a three-stage registration process employing a rigid, affine and a deformable transform based on free-form deformations using cubic B-Splines [25]. Each stage was initialized from the previous solution. We also investigated different similarity metrics for the pairwise registration (normalized cross correlation (NCC), mean squared difference (MSD) and mutual information (MI)) [26] and evaluated their impact on the accuracy of the segmentation results. The parameters used in the pairwise multi-atlas registration are summarized in Table 2. All registrations were implemented using the open source Elastix registration software package [27]. The labels associated to each atlas were propagated to the reference image using the spatial transformation obtained from the final registration stage. We also evaluated the influence on the segmentation accuracy of three label fusion techniques (majority voting [28] (MV), global normalized cross correlation (GNCC) [29] and local normalized cross correlation (LNCC)) [30] as implemented in NiftySeg [31]. For the latter two fusion techniques, the impact of the hyperparameters k (kernel size) and r (number of highest ranked atlases used) was assessed.

### 2.5. Dynamic Registration Framework

**Figure 2:**
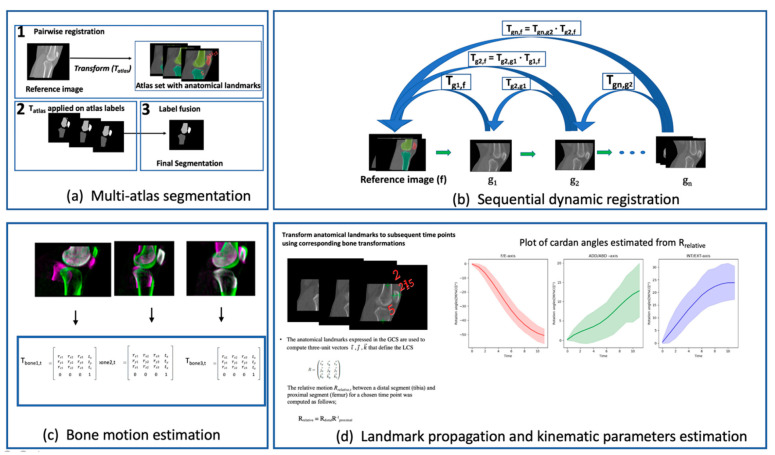
A general overview of the workflow for obtaining in vivo kinematics of bony structures. (**a**) shows the 3-step multi-atlas segmentation stage for obtaining segmentations of the reference image and propagation of anatomical landmarks. (**b**) shows the sequential dynamic registration workflow, each bone in the first time point of the dynamic sequence (g1) was aligned to the corresponding bone in the reference image (f) by the transformation (T_g1,f_) via a rigid registration. The registration between the second time point (g_2_) and the reference image was initialized with the previous transformation to obtain the transformation T_g2,f_. Subsequent time point registrations followed the same procedure. (**c**) shows an overlay of the registered bones along with transformation matrices (T_bone,t_) from which motions are estimated for each bony structure. (**d**) shows the propagation of the anatomical landmarks from the reference image to other time points using the corresponding bone transformations. Local coordinate systems (bone embedded reference frames) are defined using these landmarks. Cardan angles are estimated from unit vectors constructed using the local coordinate system to generate kinematic plots.

Motion estimation in the dynamic sequence was achieved through rigid registration in which computation of the similarity was limited to the bone of interest and its immediate vicinity. The multi-atlas segmentation approach was applied to the static reference 3DCT dataset using atlas images priorly obtained and corresponding to different subjects. The segmented reference images served as regions of interest for the rigid registration of each bone to its equivalent in the dynamic sequence. The segmented bones were dilated with a kernel radius of 3 voxels to ensure neighboring regions would be considered during the registration process. MSD was chosen as the similarity metric for this intrasubject monomodal registration because it yielded accurate results and was the least computationally demanding. We implemented a sequential intensity-based registration whereby subsequent registrations were initialized with the results of the previous registration (Figure 2II). A series of rigid transformation matrices (T_bone,t_) were obtained for each bone of interest and for each time point (t). These transformation matrices aligned each bone in the reference image to its corresponding position in the dynamic sequence. The general workflow of our proposed approach is depicted in Figure 2.

### 2.6. Landmark Propagation and Kinematic Parameters Estimation

Anatomical landmarks from the atlases were propagated onto each of the bones of interest in the reference images, using the spatial transformation obtained from the final registration stage of the MAS step. A majority voting was done to decide the winning landmark, where each landmark votes based on the local-normalized cross-correlation (LNCC) of the registered atlas to the given target at that location. Propagation of the anatomical landmarks to subsequent time frames was then performed using the estimated transformation matrices of the dynamic registration step. With these landmarks expressed in the global coordinate system (GCS) of the CT, we computed three-unit vectors, $\vec{i}$, $\vec{j}$, $\vec{k}$, to define bone embedded reference frames for each time frame. Orientation of the axis of the reference frames followed ISB recommendations [32,33].

The relative motion *R_relative,t_* between a distal segment (tibia or trapezium) and proximal segment (femur or 1st metacarpus) for a chosen time point was computed as follows;

(2) $$R_{realative,t}=R_{distal,t\;}R_{proximal,t}^{-1}$$

where *R* is a 3 × 3 rotation matrix constructed from the three-unit vectors as in Equation (3)

(3) $$R=\left[\begin{array}{ccc} i_{x} & i_{y} & i_{z}\\ j_{x} & j_{y} & j_{z}\\ k_{x} & k_{y} & k_{y} \end{array}\right]$$

Cardan angles were then subsequently extracted from results of (2) using a ZXY sequence for the thumb base and ZYX for the knee joint.

### 2.7. Validation

The MAS pipeline was validated by a leave-one-out cross-validation (LOOCV) experiment for each joint, in which data from one subject was taken as target, while the remaining were used as atlases. Success of the segmentation was evaluated using overlap and distance measures. Overlap measures consisted of false positive error (FP) and false negative error (FN) volume fractions as well as Dice coefficients (DC) [34],

(4) $$DC\left(A,B\right)=\frac{2\left|A\cap B\right|}{\left|A\right|+\left|B\right|}\;$$

(5) $$FP\;\left(A,B\right)=\frac{\left|B\backslash A\right|}{\left|B\right|}$$

(6) $$FN\;\left(A,B\right)=\frac{\left|A\backslash B\right|}{\left|A\right|}$$

where *A*, represents the ground truth (manual) binary segmentation and *B* represented the segmentation obtained by MAS. In addition, Euclidean distance maps of the ground truth manual segmentations and the surface of the corresponding segmentation obtained from the atlas-based method, were used to compute the Hausdorff distance [34]. Equation (7) shows the definition of the Hausdorff distance.

(7) h(A,B)=max{dist(A,B),dist(B,A)},

where

(8) $$dist\left(A,B\right)=\underset{x\in A}{\max}\underset{y\in B}{\min}\left|\left|x-y\right|\right|$$

We quantified the impact of introducing MAS in the dynamic registration workflow. We used the 3D Scale Invariant Feature Transform (SIFT) [35] to automatically detect a set of corresponding landmarks between the reference image and the moving image. The landmarks were checked manually to ensure an accurate and even distribution of points across all bones of interest. The Target Registration Error (TRE) was then computed as the distance between the landmarks detected on the moving image and the landmarks of the reference image transformed using results of the registration. We compared the TREs of our proposed approach to those obtained using expert manual segmentations as well as a direct B-Spline deformable registration of the whole image, initialized from a rigid + affine registration without segmentation.

Kinematic parameters obtained via our automated anatomic landmark detection were compared to those estimated using manually defined landmarks (obtained from the 3 different readers). Bland-Altman plots were created to show differences in kinematic parameters estimated with our proposed approach to that obtained using the mean of all readers as an approximation of the ground truth. We computed absolute agreement intraclass correlation coefficients (ICCs) under a two-way mixed effects model [ICC(2,*k*)] [36] to compare kinematic parameters obtained by the automated algorithm and those obtained using manually identified landmarks by the three human readers.

### 2.8. Statistical Analysis

Statistical analysis was performed using Statistical Package for Social Sciences (SPSS v23, IBM Corp, Armonk, NY, USA). We analyzed the influence of the choice of metric (NCC, MI, MSD) for the MAS registration as well as the impact of the different label fusion techniques (LNCC, GNCC, MV). Data distribution was checked using a Shapiro-Wilk test for normality [37]. Non-parametric tests were chosen since not all variables were normally distributed. To compare the fusion techniques, we used a non-parametric Friedman test for repeated measures. When the Friedman test was statistically significant, a post-hoc Wilcoxon signed-rank analysis was performed. Furthermore, the Wilcoxon signed rank test [38] was used to check for statistical significance between the mean TRE obtained by the proposed approach and the baseline method (*p* = 0.05). The distribution of the landmark identification error in the leave-one-out experiments was analyzed using descriptive statistics (median and maximal error) and box plots.

## 3. Results

### 3.1. Multi-Atlas Segmentation

**Figure 3:**
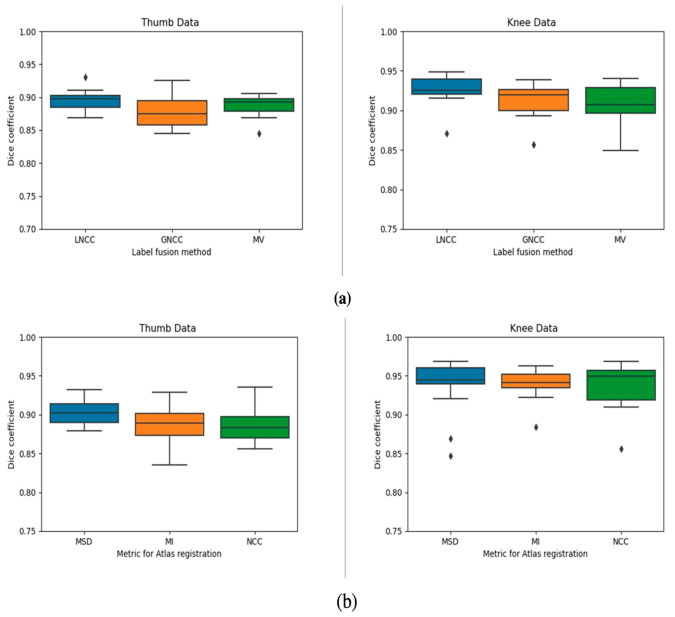
(**a**) Box plots of label fusion techniques against Dice coefficient for the two joints. These results are generated using MI as the similarity metric for the pairwise registrations. Parameters for LNCC were k = 5, r = 3 and for GNCC r = 3. (**b**) Plots of similarity metrics (used in the pairwise registration between atlases and images to be segmented) against Dice coefficient for the two joints.

Figure 3 summarizes the results of the segmentations using overlap measures. We successfully segmented the bones of interest for both the knee and thumb dataset resulting in mean Dice coefficients above 0.90. No significant differences were observed between the three investigated similarity metrics (X2 = 4.7, *p* = 0.09). We therefore chose MSD in subsequent experiments because of the low computational complexity.

Concerning the label fusion, the Friedman test showed significant differences between the label fusion techniques. Post-hoc Wilcoxon signed rank tests revealed that LNCC was significantly better than GNCC for all joints (*p* < 0.001).

**Figure 4:**
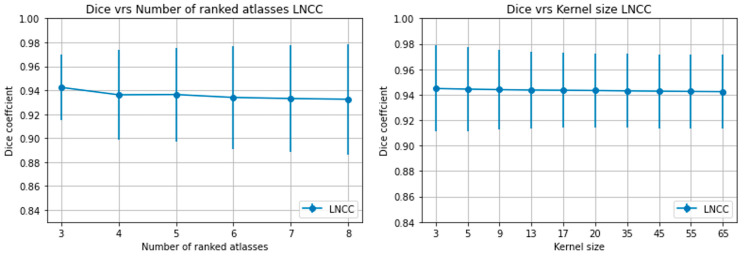
(**a**) Plot of Dice coefficient against number of highest ranked atlases (r) for a fixed kernel size = 5 voxels and (**b**) dice coefficient against kernel size (k) for a fixed r = 3 for the knee.

**Figure 5:**
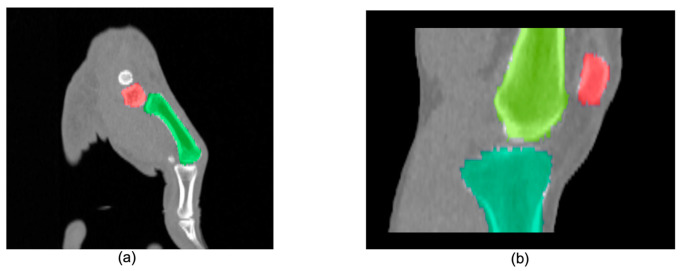
Segmentation result of our multi-atlas multi-label segmentation for (**a**) thumb base and (**b**) knee joint.

The hyperparameters, kernel size (k) and the number of highest ranked atlases (r), had a marginal impact on the Dice score (Figure 4). Consequently, we selected LNCC with k = 5 and r = 3 to obtain the final automatic segmentations. Table 3 summarizes the quantitative results of these experiments. An example of the volume rendered segmentation for the two joints using LNCC (k = 5, r = 3) is shown in Figure 5.

### 3.2. Dynamic Registration

**Figure 6:**
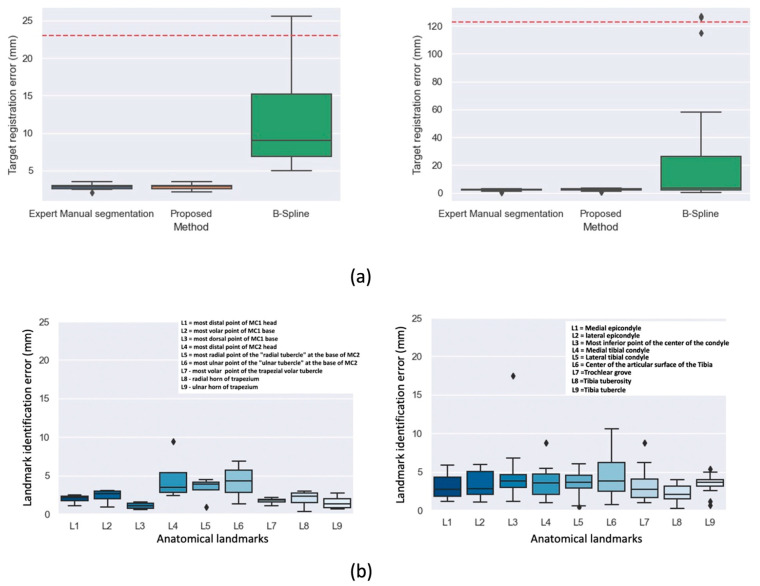
(**a**) Box plots showing TRE results of the piecewise rigid dynamic registration step for thumb base (top-left, *n* = 5) and the knee joint (top-right, *n* = 10). Results are shown for the expert manual segmentation approach, our multi-atlas guided approach (MAS) and a deformable registration (B-Spline). Dashed red lines indicate TRE for unregistered images (**b**) landmark identification error of the automatic anatomic landmark identification approach compared to the mean of all readers across 9 landmarks for thumb base (bottom-left) and the knee joint (bottom-right). The names of the anatomical landmarks are shown as inserts on the graphs.

The box plots in Figure 6a show the TRE results of the dynamic registration step. Introducing our MAS approach in the dynamic registration framework successfully registered the dynamic sequences and performed on par (Wilcoxon 2-tailed ranked test; *p* = 0.51) with a manual segmentation-guided approach. As a comparison, we also evaluated the TRE of a direct deformable registration, without prior segmentation of the bones. The large values for the TRE obtained indicate the registration often failed, resulting in poor overlap and confirming the challenging nature of the problem.

### 3.3. Landmark Propagation

Concerning the landmark identification accuracy, Figure 6b summarizes the landmark identification error of the automatic algorithm to the mean of all readers taken as ground-truth. The femur center diaphysis and tibia center diaphysis landmarks used for estimating the femoral and tibial axes were omitted in the landmark identification error plots of Figure 6b. These points were eliminated because the images had to be cropped at those areas due to image artifacts. Consequently, the deformable registration employed in the final stage of the MAS mapped these landmarks outside the image regions for some subjects. While this had no impact on the computation of the bone-embedded reference frames, it resulted in high landmark identification errors. We therefore replaced these two landmarks with the most inferior point at the center of the condyle and center of the articular surface of the tibia. Each graph shows the distribution of distance errors of the landmarks for the leave-one-out test images, with median errors below 5 mm for all landmarks on both the thumb base and knee joint. The highest values of the median error for the knee are found for the most inferior point of the center of the condyle (L3) and center of the articular surface of the tibia (L6) with median errors of 4.8 mm and 4.3 mm respectively. For the thumb base, median errors of 4.7 mm and 4.2 mm were observed for the most distal point of the second metacarpal (L4) and the most ulnar point of the ulnar tubercle at the base of the second metacarpal (L6).

### 3.4. Kinematic Parameters

**Figure 7:**
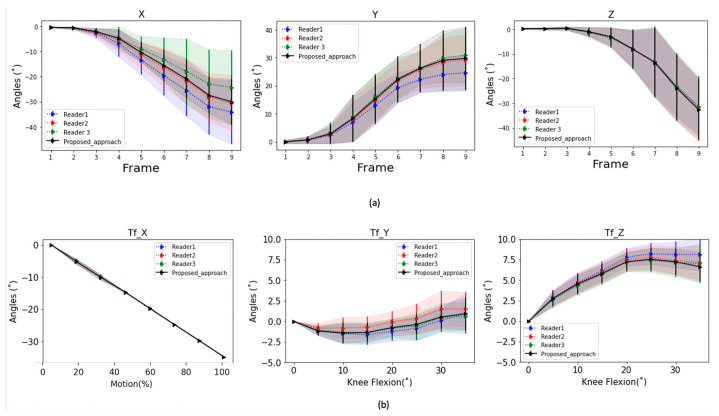
(**a**) 1st Metacarpal bone motion (cardan angles) showing an opposition movement of the thumb from neutral to full opposition. The plots show results using the proposed approach compared to using manual landmarks identified by three readers. X represents the Flexion (−)/Extension (+) axis, Y is the Adduction (−)/Abduction (+) and Z represents the Internal (+)/External (−) rotation axis; (**b**) Tibiofemoral (Tf) joint motion (cardan angles) obtained in leave-one-out validation on 10 subjects for the first 30° of knee flexion. The plots show results using the proposed approach compared to using manual landmarks identified by the three readers. Shaded regions represent 95% Confidence Interval over all subjects. (**a**) Tf_X represents the Flexion (−)/Extension (+) axis, Tf_Y represents Adduction (−)/Abduction (+) axis and TF_Z represents Internal (+)/External (−) rotation axis.

Performance of the proposed algorithm in estimating kinematic parameters is summarized in Figure 7a for the thumb base and Figure 7b for the knee joint. Results of cardan angles using our proposed approach are plotted together with results from manually identified landmarks of the 3 readers on the same graph. Shaded regions represent 95% Confidence Interval from the leave-one-out experiments.

**Figure 8:**
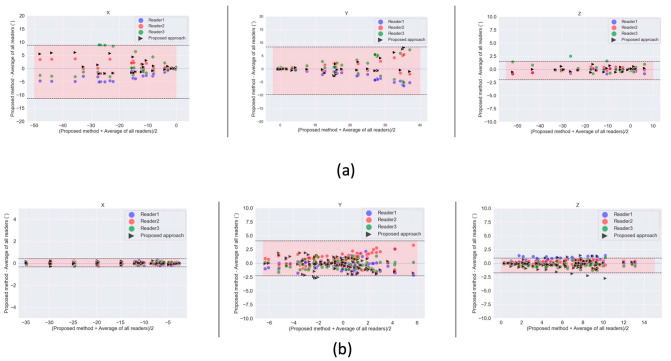
Bland Altman plots showing the limits of agreement between our proposed approach for kinematic parameter estimation (cardan angles) and a manual landmark identification (by three readers) approach for (**a**) thumb base; (**b**) knee. The mean of landmarks identified by the three readers is compared to our multi-atlas segmentation and landmark propagation approach. Shaded regions represent the limits of agreement of the three readers combined.

The Bland-Altman plots in Figure 8 also show the limits of agreement between our proposed approach and the manual approach for both the thumb base and knee joint. As in Figure 6b, results shown in Figure 8 are computed against the mean of all 3 readers. Our proposed approach produces kinematic parameters which fall within the limits of agreement of all three readers as is evident in Figure 8. Intraclass correlation (ICC) between cardan angles from the algorithm and results from expert manual landmarks ranged from 0.72 to 0.99 for all joints across all axes as detailed in Table 4.

### 3.5. Discussion

We proposed an automated method for kinematic assessment of bony joint structures, based on multi-atlas segmentation of bony structures and landmark propagation. We evaluated this on a dataset of dynamic CT acquisitions of the thumb base and knee joint. Experiments were conducted to investigate the influence of the similarity metric in the MAS registration step, and we observed no significant differences in the choice of metric, allowing us to use MSD for our study. In case the dynamic sequence is from a different modality as the atlas (CBCT, MRI), alternative metrics such as NCC and MI will need to be tested.

The choice of the label fusion technique had an influence on the accuracy of the final segmentation, with LNCC performing better than the other fusion techniques. This can be attributed to the fact that LNCC computes a local normalized cross-correlation similarity using a 3D kernel and selects the best matching atlases based on this to be used in a majority vote. This captured the spatially varying nature of the registration accuracy and (locally) ignore poorly registered atlases that might misguide the final segmentation result. Our findings are in line with the work of Ceranka et al. [26] and Arabi et al. [39], both showing a better performance of the LNCC label fusion technique. The impact of both r and k on LNCC was marginal. 

The impact of the number of atlases was not investigated in this study. Ceranka et al. [24] performed an analysis on the influence of the number of atlases on the quality of the segmentation of skeletal structures in whole-body MRIs and only found a marginal improvement above six atlases. The number of atlases used in this current study (*n* = 4 for thumb, *n* = 9 for knee) yielded Dice coefficients of 0.90 ± 0.01 for the thumb and 0.94 ± 0.02 for the knee. We believe that increasing the number of atlases for the thumb may increase segmentation accuracy further.

Our MAS approach with the best label fusion technique (LNCC, k = 5, r = 3) facilitated the segmentation of reference images, which were introduced in the dynamic registration framework. Accuracy of the dynamic registration workflow was evaluated using TRE. We compared the TRE results of our approach with results obtained using manually segmented images and observed no significant difference with our proposed approach (*p* = 0.51). Conversely, direct deformable registration of the joint images, without prior segmentation, led to mean errors around 10 mm and failed registrations (outliers). 

The use of anatomical landmark propagation to define local bone-embedded reference frames further justifies the need for a multi-atlas segmentation approach for the segmentation of bones of interest. The spatial transformation obtained from the MAS automates the detection of anatomical landmarks in reference images. These landmarks can be propagated across the entire dynamic sequence automatically using transformations obtained from the dynamic registration step. Moreover, metrics based on changes of bone landmarks distance over time such as tibial-tuberosity trochlear groove [40] (used for subject with patella instability) can be extracted using the same approach. This can facilitate orthopedic diagnosis and surgical planning. Our automated landmark approach for estimating kinematics performed on par to the manual identification of landmarks by three independent readers, as shown by the Bland-Altman plots with mean differences falling within the limits of agreement of the readers across all axes for both joints. Beside cardan angles, other parameters such as bone surface contacts can be calculated from the obtained transformation matrices [41,42]. Our proposed approach uses a set of annotated datasets (atlases) but requires a reduced number (*n* = 5, *n* = 10 for thumb and knee) as it belongs to the group of methods that make use of image registration. This contrasts with machine learning algorithms, [22], which rely on a significant amount of annotated data in training to yield good results. 

Similar algorithms to the proposed method both in terms of multi-atlas methodology and anatomical landmarks identified are presented in [43,44]. Our current study however demonstrated the generalizability of the proposed approach to other joints by applying it on dynamic CT of the knee and thumb. In [44], the authors proposed an algorithm for automatic anatomical measurements in the knee based on landmarks on CBCT images. A comparison between our approach and [44] can only be made on the knee data. Taking into consideration corresponding anatomical landmarks, L7 in our work corresponds to FT1 in [44], L8 corresponds to TT1, L5 to TP8 and L4 to TP9. Other potential corresponding points were excluded in the error analysis of [44] because they were not associated with any specific anatomical features. The average LDE of available points for comparison is 3.75 mm for [44] against 4.27 mm in our work. In general, our approach reaches comparable accuracy to previously reported algorithms for musculoskeletal applications [45,46] which reported median errors from ~2.5 to ~6 mm. Furthermore, results obtained from the kinematic analysis are within the limit of agreements of the three independent readers. 

A potential limitation of the proposed approach is the computationally expensive pairwise registrations needed in the MAS step. Segmentation of a single subject using *n* = 10 atlases was completed in 40 min on a 2.6 GHz Intel Core i7 16 GB ram computer. To speed up this step, approaches which involve selecting relevant atlases as opposed to a registration with all available atlases can be considered [47,48,49]. The use of the capabilities of GPU processors have also been proposed to help accelerate the registration step [50]. 

Another potential limitation of this study is the definition of ground-truth anatomical landmarks on the atlas dataset. The mean of the three readers and error analysis was also done with respect to the mean of all the readers. There is however the potential of introducing errors if one of the readers’ landmarks are poorly defined. A potential solution is to propose a consensus framework like that proposed in [51], for combining segmentations. 

Furthermore, this study only involved 15 healthy subjects which limits making detailed inferences from the obtained kinematic parameters. The homogenous nature of the study population (in terms of age and health status) also means the atlases were constructed with bones that do not exhibit unique or pathological morphology. Processing a new subject with such morphological variants may limit the success of the MAS step as well as the anatomic landmark propagation. Nonetheless, the deformable registration stage introduced in the workflow could compensate for some of the variations in morphology. It is also likely that manual landmark identification would be equally challenging in such situations.

### 3.6. Conclusions

Quantitative imaging modalities are becoming increasingly useful in understanding and evaluating MSK conditions, with dynamic CT being a promising tool [52]. The 4D MSK images generated from this technique are however not intuitive and in general require automated image analysis procedures to extract quantitative estimates of joint kinematics. We proposed a multi-atlas multi-label bone segmentation and landmark propagation approach and used it as an input for the kinematic analysis of dynamic CT images of two joints. Our method performed on par with commonly used approaches requiring manual segmentation and landmark identification. As such, it contributes to the build-up of an automated workflow for the post-processing of dynamic CT MSK images. Such quantitative assessment could increase the clinical value of radiologic examinations as it adds a functional dimension to morphological data.

Future studies will include reducing the time for the computationally expensive pairwise registrations of the MAS and the dynamic registration step by means of GPU implementation. The introduction of deep learning and conventional machine learning methods will also be considered using results of this study as annotated data. 

## Figures and Tables

**Table 1:** Overview of scan parameters for the dynamic and static acquisitions.

	Dynamic Acquisition	Static Acquisitions
**Knee**		
Tube Voltage	80 kV	120 kV
Tube current	50 mA	80 mA
Tube rotation time	0.28 s	0.28 s
Reconstructed slice thickness	2.5 mm	2.5 mm
Field of View	500 mm	500 mm
Collimation	256 × 0.625 mm	256 × 0.625 mm
Dose length product	107.91 mGycm	23.06 mGycm
* CTDI	6.74 mGy	1.44 mGy
**Thumb**		
Tube Voltage	80 kV	120 kV
Tube current	50 mA	80 mA
Tube rotation time	0.28 s	0.28 s
Reconstructed slice thickness	1.25 mm	1.25 mm
Field of View	300 mm	300 mm
Collimation	192 × 0.625 mm	192 × 0.625 mm
Dose length product	156.45 mGycm	19.58 mGycm
CTDI	13 mGy	1.63 mGy

* Computed tomography dose index.

**Table 2:** Registration parameters used for the multi-atlas registration.

Parameter	First Stage	Second Stage	Final Stage
Similarity Metric	(MSD/MI/NCC) *	(MSD/MI/NCC) *	(MSD/MI/NCC) *
Regulariser	/	/	Bending energy
Transform	Rigid	Affine	B-Spline
Multi Resolution levels	4	4	4
Number of histogram bins used for MI	32	32	32
Sampler	Random	Random	Random
Max iterations	2000	1000	1000
Number of samples	2000	2000	2000
Optimizer	Stochastic Gradient Descent	Stochastic Gradient Descent	Stochastic Gradient Descent

* All three metrics were investigated.

**Table 3:** Segmentation evaluation criteria results (Mean ± SD) over the leave-one-out cross-validation for the 2 joints using LNCC (k = 5, r = 3).

Joint	Dice Score	FP	FN	Mean Surface Distance (mm)	Max Surface Distance (mm)	SD Surface Distance (mm)
**Thumb**	0.90 ± 0.01	0.08 ± 0.02	0.14 ± 0.03	0.53 ± 0.05	4.89 ± 1.25	0.68 ± 0.05
**Knee**	0.94 ± 0.02	0.05 ± 0.02	0.06 ± 0.02	0.42 ± 0.16	4.91 ± 1.13	0.66 ± 0.18

FP = false positive error fraction, FN = false negative error fraction.

**Table 4:** ICCs of cardan angles obtained by expert readers and by the proposed automated workflow (Auto) for the three axes for the thumb and knee.

Thumb	* AUTO		
	X	Y	Z
Reader 1	0.99	0.99	0.99
Reader 2	0.95	0.94	0.99
Reader 3	0.92	0.94	0.99
Reader AVG	0.95	0.97	0.99
**Knee**	X	Y	Z
Reader 1	0.99	0.72	0.96
Reader 2	0.99	0.76	0.95
Reader 3	0.99	0.83	0.94
* Reader AVG	0.99	0.82	0.96

* Auto: the proposed automated workflow, * Reader AVG: the average of all three reader.

## Data Availability

Data supporting this study can be obtained by contacting the corresponding author.

## Supplementary Materials

The following are available online at https://www.mdpi.com/article/10.3390/diagnostics11112062/s1, Video S1: dynamic CT volume render thumb, Video S2: dynamic CT of Knee.
