# Supplementary figures and images for: Automated Motion Analysis of Bony Joint Structures from Dynamic Computer Tomography Images: A Multi-Atlas Approach
Source: Diagnostics (Basel). 2021 Nov 7;11(11):2062. doi: 10.3390/diagnostics11112062 (PMC8621122; doi:10.3390/diagnostics11112062)

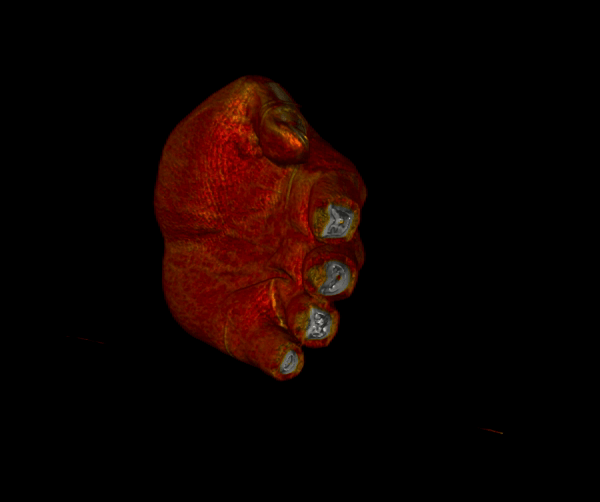

Supplement: Supplementary file 1 [file diagnostics-11-02062-s001.zip › Supplementary_Diagnostics/dynamic CT volume render thumb.gif]

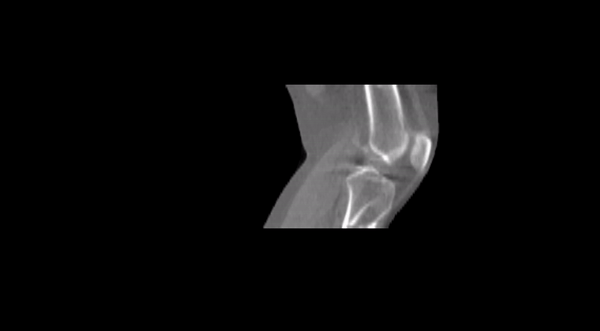

Supplement: Supplementary file 1 [file diagnostics-11-02062-s001.zip › Supplementary_Diagnostics/dynamic CT of Knee.gif]
